# A Study of the Experience and Understanding of Religious Obsessive-Compulsive Disorder as It Applies to Pastoral Ministry, in a Sample of Local Clergy.

**DOI:** 10.1192/bjo.2026.11138

**Published:** 2026-06-30

**Authors:** Craig Thompson

**Affiliations:** Tees Esk and Wear Valleys NHS Foundation Trust, Darlington, United Kingdom

## Abstract

**Aims:**

A number of subtypes of obsessive-compulsive disorder (OCD) have been described, one of which is religious OCD (ROCD), also referred to as Scrupulosity Disorder. Whilst there is an expanding literature which investigates the link between OCD and religion, there is very little research which examines the impact of clergy attitudes and intervention upon a sufferer of ROCD. The study aims to begin the task of developing an understanding of how those in Christian pastoral ministry in the UK typically view ROCD, how they are likely to deal with it in someone for whom they have pastoral care, and whether such interventionsare compatible with current models of clinical care.

**Methods:**

The study involved conducting in-depth interviews with six respondents working professionally in pastoral ministry. The respondents were interviewed using a questionnaire comprising a series of open questions. The initial phase of the interview was designed toencourage discussion as to how a respondent might react to someone with typical ROCD symptoms, having had such explained to them. The nature of ROCD as a psychiatric diagnosis was then explained, including some cognitive theory and a description of typical treatment. The remainder of the interview concerned itself with exploring respondents’ views on these matters.

**Results:**

There was general consistency of thought between correspondents; all were willing to give credence to a psychopathological attribution of ROCD and the implication that a psychological approach is useful in its management. All were clear that an empathetic and sensitive approach is necessary and that a response which conveys a sense of judgement is to be avoided. Recommendations were generated which would broadly be viewed as psycho education from a clinical standpoint: reflection on the nature of God; an explanation that obsessions do not confer culpability nor are they indicative of an underlying spiritual or moral deficit; an exploration of the dissonance created by the sufferer’s unsuccessful attempts to resist compulsive behaviour.

**Conclusion:**

The principal source of conflict between pastoral care and treatment occurs when a minister seeks to offer reassurance to combat the doubts and anxieties of a sufferer. Conversely, respondents’ recommendations are clearly well suited to pastoral intervention, being of a theological nature. It is here, with the ‘theological examination’ of a sufferer’s negative appraisal of obsessions, that the minister might best use the authority invested in him/her, rather than by the giving of permission to follow the edicts of the therapist.

